# Persistent lymphopenia after kidney transplantation: increased mortality and decreased homeostatic mechanisms

**DOI:** 10.3389/fimmu.2025.1605794

**Published:** 2025-06-19

**Authors:** Yun Liang, Byron Smith, Walter Park, Zhihao Li, Ahmed Abdelrheem, Esma Kesik, Mateo Velasquez Mejia, Kevin Pavelko, Erik Jessen, Tambi Jarmi, Girish K. Mour, Sumi S. Nair, Mark D. Stegall

**Affiliations:** ^1^ Department of Surgery, Mayo Clinic, Rochester, MN, United States; ^2^ Division of Clinical Trials and Biostatistics, Mayo Clinic, Rochester, MN, United States; ^3^ William J. von Liebig Center for Transplantation and Clinical Regeneration, Mayo Clinic, Rochester, MN, United States; ^4^ Immune Monitoring Core, Mayo Clinic, Rochester, MN, United States; ^5^ Department of Immunology, Mayo Clinic, Rochester, MN, United States; ^6^ Division of Computational Biology, Mayo Clinic, Rochester, MN, United States; ^7^ Division of Transplantation Medicine, Mayo Clinic, Jacksonville, FL, United States; ^8^ Division of Nephrology, Mayo Clinic, Scottsdale, AZ, United States

**Keywords:** kidney transplant, lymphopenia, mortality, induction, immunosuppression

## Abstract

**Introduction:**

Kidney transplant (KTx) recipients commonly develop transient lymphopenia due to treatment with alemtuzumab, rabbit anti-thymocyte globulin (rATG) or other conditions. However, persistent lymphopenia lasting for years has not been studied in detail. The goal of this study was to determine the prevalence of persistent lymphopenia, evaluate its association with mortality and investigate possible mechanisms by which it occurs.

**Methods:**

We retrospectively studied peripheral blood lymphocyte and leukocyte counts in 7307 adult, solitary renal transplant recipients transplanted between 1/2006 to 12/2020.

**Results:**

While leukocyte counts generally remained within the normal range, lymphocyte counts 3 years after KTx were below normal in 31% (compared to 14% pretransplant and 54% at 1 year). Increasing severity of lymphopenia at 3 years was associated with increasing risk of mortality. The major risk factors for lymphopenia at 3 years were: receiving alemtuzumab or rATG for induction or the treatment of rejection, increasing recipient age, pretransplant dialysis, a low lymphocyte count pretransplant, and having a prior kidney transplant. Mass cytometry immunophenotyping at 3 years showed that B cells, NK cells and all T cell subsets (CD4, CD8, naïve, memory, etc.) decreased with decreasing lymphocyte counts. This included fewer recent thymic emigrants, naïve T cells, and stem-cell like memory T cells (T_SCM_), suggesting an impaired homeostasis of peripheral T cells. PD-1 expression was increased with decreasing lymphocyte counts in T and B cells and in most T cell subsets including CD4 T_SCM_, CD4 and CD8 naïve cells, and CD4 recent thymic emigrants.

**Discussion:**

We conclude that persistent lymphopenia might be partially due to impaired homeostatic mechanisms in T, B and NK cells and might be a simple, useful biomarker for individualizing patient management.

## Introduction

Kidney transplant (KTx) recipients commonly develop transient reductions in peripheral blood lymphocyte counts. In the US, approximately 80% of KTx recipients receive lymphocyte depleting agents (alemtuzumab or rabbit anti-thymocyte globulin (rATG) for induction therapy and others receive them for the treatment of rejection (1). Several studies have characterized the early kinetics of immune reconstitution after induction with these agents suggesting that B and NK cells return rapidly, while T cell counts may take 1–2 years to return to normal (1). In addition to these agents, Cytomegalovirus (CMV) infection, the use of antimetabolites such as mycophenolate mofetil, and concomitant medications such as valganciclovir can contribute to lymphopenia.

Most studies of lymphopenia after transplant have focused on the early post-transplant time period and data on the prevalence of lymphopenia years after kidney transplantation are lacking. We hypothesized that persistently low lymphocyte counts years after KTx would correlate with long-term mortality. One advantage of using lymphocyte counts as a biomarker is that they are part of routine blood count assays. Additionally, severe lymphopenia may be modifiable through avoidance of alemtuzumab or rATG and minimization of post-transplant medication such as mycophenolate mofetil.

The goal of this study was to examine the kinetics of lymphocyte counts in the first few years after KTx in the current era of immunosuppression in a large, multicenter cohort. We aimed to determine the prevalence of persistent lymphopenia, risk factors and impact on subsequent mortality. We also aimed to examine specific lymphocyte subsets affected to determine possible mechanisms of persistent lymphopenia.

## Methods

### Patient selection

Using a protocol approved by the Mayo Clinic Institutional Review Board, we retrospectively studied 7307 adult solitary KTx recipients transplanted between 1/1/2006 to 12/31/2020 at the three Mayo Clinic sites in Minnesota, Florida, and Arizona. Last follow-up date was 2/27/2025. Retransplants were included (i.e. recipients who were transplanted during the study period who had a prior KTx before the study period). For those who received more than one kidney transplant during the study period, only details related to their first transplant episode were included. Recipients of another solid-organ transplant were excluded. Recipients whose renal failure was attributed to either multiple myeloma or amyloidosis also were excluded because most had received either a hematologic stem cell transplant or other chemotherapy that might profoundly affect their lymphocyte counts. Patients were administratively censored when follow-up was no longer available, such as moving internationally or no longer having access to a translator. Lost to follow up was defined as not having information regarding their status (alive, dead, graft status) as of 2/27/2024 regardless of their original date of transplant (i.e. we had data on the patient updated within the past year).

Total peripheral blood leukocyte and lymphocyte counts were reported as part of an automated complete blood count and were obtained as standard of care management at baseline, at 4 months, and annually post-KTx. The assays are performed by College of American Pathologists (CAP) and Clinical Laboratory Improvement Amendments (CLIA) certified clinical laboratories at Mayo Clinic Arizona, Florida or Minnesota and the results recorded into the electronic medical record. As previously described (2), all other information related to the transplant episode and subsequent clinical outcomes, including graft status and patient status, were obtained using a combination of review of the electronic medical record, single center data from the Scientific Registry of Transplant Recipients, and direct telephone or internet contact. Data were stored in a dedicated transplant database at Mayo Clinic.

Protocols for CMV prophylaxis and immunosuppression are presented in Supplementary Methods. In general, recipients > 65 years of age received basiliximab (Simulect™) induction unless sensitized or a retransplant. All others received either alemtuzumab (Campath™) or rabbit anti-thymocyte globulin (rATG, Thymoglobulin™). The vast majority of recipients had maintenance immunosuppression including tacrolimus, mycophenolate mofetil, and ± prednisone.

### Cytometry by time of flight

A subset of 84 recipients underwent immunotyping via CyTOF three years post-KTx. This cohort was selected to represent a diverse range of lymphocyte counts, CMV serostatus, age, and death with a functioning graft (DWFG), while excluding retransplants and those treated for acute cellular rejection within the first three years. Detailed methods for CyTOF processing and gating strategies, including a complete list of markers and phenotypes examined, are provided in the Supplementary Methods.

### Statistical analyses

Continuous variables were summarized using the mean and standard deviation or the median and inter-quartile range where appropriate. Categorical variables are summarized using count and percentage. Differences between groups were tested using ANOVA or the Kruskal-Wallis Test for continuous variables or chi-square tests for categorical variables. Correlations between lymphocyte count and cell subset proportions and absolute counts utilized Pearson correlation. Variable associations with low lymphocyte count were tested using univariate and multivariable logistic regression.

The primary endpoint was time to death with a functioning graft (DWFG). Incidences were summarized using the Kaplan-Meier method and tested using the Log-rank test. Univariate and multivariable Cox regression models were used to look at the association of baseline and post-transplant variables with mortality post-transplant. Linearity of all variables were assessed with psplines from the ‘survival’ package in R. These splines are also used to demonstrate changes in the relationship between lymphocyte count and mortality post-transplant. Proportionality was tested by plotting the Schoenfeld residuals.

## Results

### Study cohort

Demographics, pre-transplant and transplant characteristics are included in **Table 1**. The mean age at the time of transplantation was 53.43 ± 13.97 years. There was a wide range of race/ethnicity including: 64.6% Caucasian/Non-Hispanic, 13.3% Black, 12.6% Hispanic, 3.8% Native American and 5.7% Other (Asian, Pacific Islanders and other self-identified). Of the total cohort, 52.5% received a deceased donor kidney, 71.9% were on dialysis at the time of transplantation and 9.8% had a prior kidney transplant.

**Table 1:** Demographics of the entire study population.

Category	Variable	Mean ± Stdev	Incidence
Demographics	Age	53.428 ± 13.974	
	Age >55		3817 (52.2%)
	Male		4357 (59.6%)
	Race/Ethnicity		
	Caucasian/Non-Hispanic		4718 (64.6%)
	Black		973 (13.3%)
	Hispanic		920 (12.6%)
	Native American		280 (3.8%)
	Other		416 (5.7%)
Pre-transplant			
	BMI	28.657 ± 5.754	
	Dialysis		5257 (71.9%)
	Diabetes		2370 (32.5%)
	CMV seronegative		2804 (38.4%)
Transplant			
	Deceased donor		3838 (52.5%)
	Induction		
	**Lymphocyte depleting**		**5512**
	Campath (Alemtuzumab)		3062 (42.0%)
	Thymoglobulin (rATG)		2450 (33.6%)
	**Non depleting**		**1851**
	Other		76 (1.0%)
	Simulect (Basiliximab)		1701 (23.3%)
	None		1 (0.0%)
	Prior kidney transplant		718 (9.8%)
	3 year white blood cell count (x106 cells/ml)	6.637 ± 2.624	
	3 year absolute lymphocyte count (x10^6^ cells/ml)	1346.36 ± 723.80	
	Rejection in the last 3 years		905 (26.9%)
	Follow-up time (years)	7.16 ± 4.32	
	Graft status		
	Active		4630 (63.4%)
	Death with Function		1741 (23.8%)
	Graft loss		936 (12.8%)
	Causes of Death		
	Cancer		188 (10.8%)
	Cardiac		132 (7.6%)
	Infection		290 (16.7%)
	Other		137 (7.9%)
	Unknown		994 (57.1%)

Bold values signify a subcategorization of the induction agents into 2 specific categories (T-cell depleting and non-depleting agents) before further summaries of the specific agents.

The mean follow-up time was 7.16 ± 4.32 years. Only 4.5% were lost to follow-up. Lymphocyte depleting induction was given to 75.6% (42.0% alemtuzumab and 33.6% rATG) and 23.3% received basiliximab. By 3 years after KTx, 26.9% had been treated for rejection including 23 who received basiliximab induction. Thus, by 3 years 5551 (77.4%) had received either ATG or alemtuzumab treatment. There were 1061 graft losses and 2010 deaths with a functioning graft (DWFG). The causes of death were: infection (16.7%), cancer (10.8%), cardiac (7.6%), other (7.9%) and unknown (57.1%). Demographics of KTx with unknown causes of death were similar to those with known causes and thus the discrepancy was largely due to lack of detailed data (data not shown).

#### Leukocyte and lymphocyte counts at baseline and post-transplant

**Figure 1:**
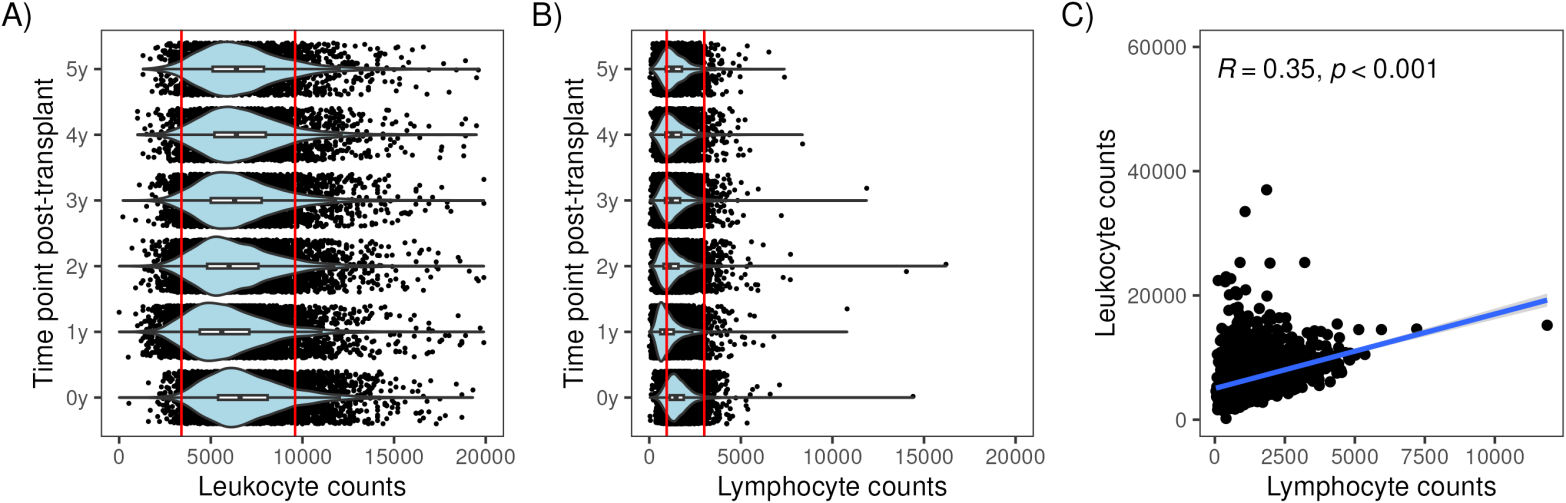
Distribution of lymphocyte and leukocyte counts at multiple time points with respect to kidney transplantation. Violin plots showing the distribution of lymphocyte counts in **(A)** and leukocyte counts **(B)** at baseline (0y) and 1 to 5 years post-transplant. Scatterplot showing the correlation between lymphocyte counts (x106 cells/ml) and leukocyte counts (x106 cells/ml) at 3 years in **(C)**.

At the time of transplant, 2% of recipients had a peripheral blood *leukocyte* count below the normal range (3400–9600 leukocytes/µl, 95% of values of the general population are within this range) and 14% had a lymphocyte count below the normal range (950–3000 lymphocytes/µl) (see **Table 2**). Post-transplant, leukocyte counts tended to remain within the normal range. In contrast, lymphocyte counts after transplant were commonly below the normal range: 1-year: 54%, 2-year: 38%, 3-year: 31%, 4-year: 30% and 5-year: 28% (**Table 2**). Lymphocyte counts were skewed toward the lower counts (**Figure 1B**) while leukocyte counts were more evenly distributed (**Figure 1A**). There was a modest correlation between peripheral blood leukocytes and lymphocytes at 3 years (R=0.35, p<0.001, **Figure 1C**) and at other time points (data not shown).

**Table 2:** Leukocyte and lymphocyte counts are various time points post-transplant..

Time	Leukocyte count				Lymphocyte count			
	N	< 3400	> 9600	3400-9600	N	< 950	> 3000	950 - 3000
Time of transplant	6709	117 (2%)	771 (11%)	5821 (87%)	7200	1037 (14%)	181 (3%)	5982 (83%)
1 year	6547	562 (9%)	445 (7%)	5540 (85%)	6332	3402 (54%)	103 (2%)	2827 (45%)
2 year	5448	272 (5%)	463 (8%)	4713 (87%)	5145	1931 (38%)	147 (3%)	3067 (60%)
3 year	4497	190 (4%)	436 (10%)	3871 (86%)	4143	1275 (31%)	113 (3%)	2692 (66%)
4 year	3207	115 (4%)	314 (10%)	2778 (87%)	2672	812 (30%)	85 (3%)	1775 (66%)
5 year	2614	97 (4%)	285 (11%)	2232 (85%)	2175	617 (28%)	68 (3%)	1490 (69%)

#### Association between lymphocyte counts and death with a functioning graft

**Figure 2:**
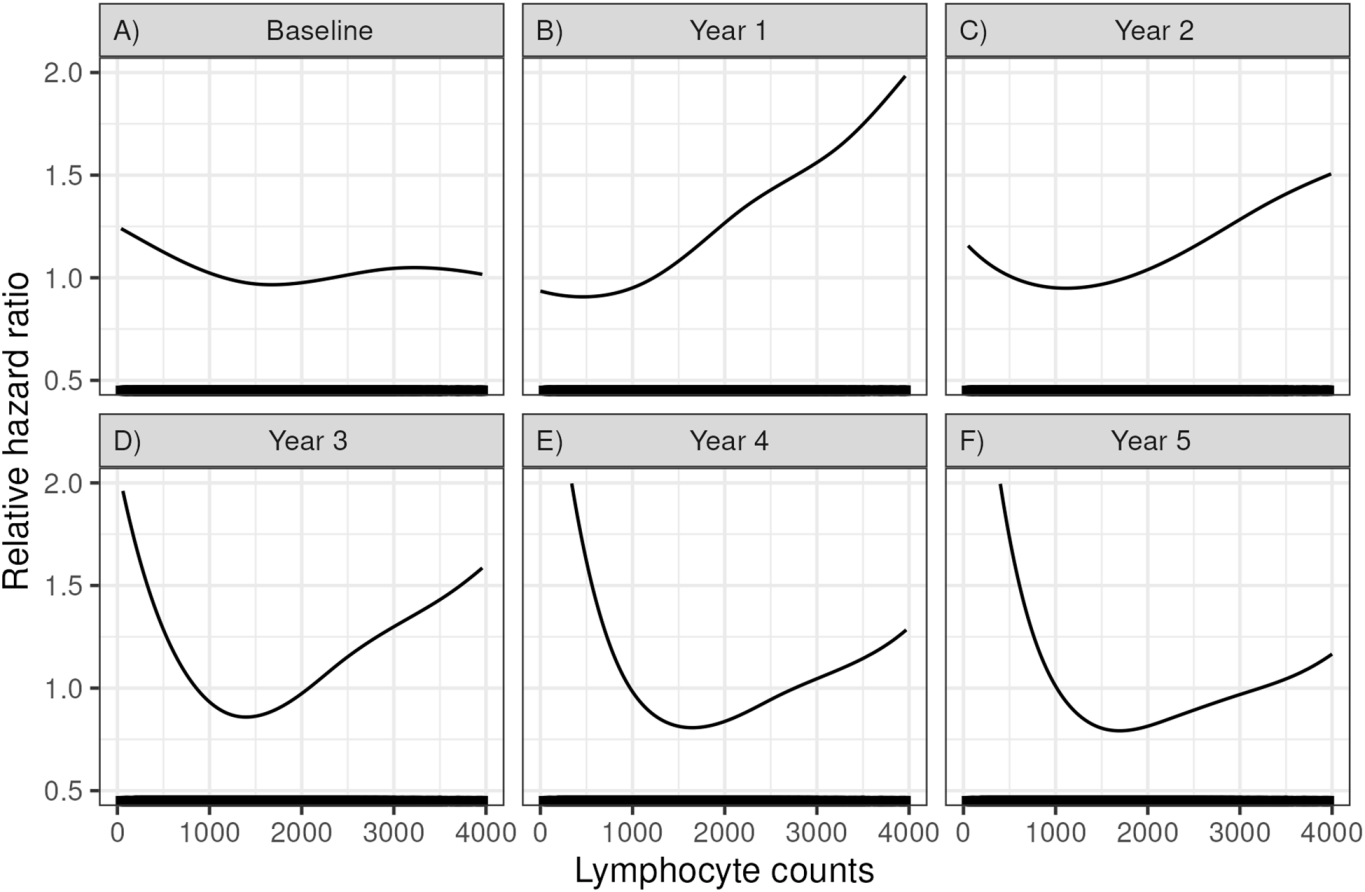
Relative hazard ratios for mortality based on a Cox regression using a spline fit for lymphocyte count at **(A)** time of transplant (baseline), **(B)** 1 year, **(C)** 2 years, **(D)** 3 years, **(E)** 4 years, and **(F)** 5 years post-transplant.

We hypothesized that a low lymphocyte count would correlate with increased rates of death with function. **Figure 2** shows how lymphocyte counts at various time points correlated with the risk of post-transplant mortality (using the pspline function, see methods). At baseline, 1 and 2 years after KTx, lower lymphocyte counts had a minimal impact on mortality. However, at years 3, 4 and 5, a progressively lower lymphocyte count correlated with a progressively higher risk of mortality. A high lymphocyte count also was associated with increased mortality, but the number of patients was small and were not considered further in our analyses.

#### Patient and clinical variables associated with 3-year lymphocyte counts

Based on the above data, we further examined lymphocyte counts at 3 years after KTx. Using lymphocyte count as a continuous variable, we performed a multivariable linear regression model involving demographics and transplant characteristics to identify factors that correlated with 3-year lymphocyte counts (**Table 3**). In the multivariable model the risk factors for lower lymphocyte counts in their order of statistical significance were: the use of rATG or alemtuzumab (either for induction or for the treatment of rejection), lymphocyte count at the time of transplant, increasing age of the recipient at the time of transplant, being on dialysis prior to transplant, or having a prior kidney transplant. Recipients with Asian/other ethnicity (compared to White, Non-Hispanic Recipients) had higher lymphocyte counts.

**Table 3:** Univariate and multivariate analyses of the recipient and transplant characteristics associated with 3-year lymphocyte count levels.

Category	Variable	Univariable		Multivariable	
		Estimate (cells/µl per value of predictor)	P	Estimate (cells/µl per value of predictor)	P
Demographics					
	Age (years)	2.05 (0.44, 3.65)	0.0124	-4.09 (-5.88, -2.30)	<0.0001
	Male	-13.69 (-58.77, 31.38)	0.5514	1.3 (-41.06, 43.66)	0.9520
	Race				
	Black	0.73 (-64.79, 66.24)	0.9827	31.37 (-34.22, 96.95)	0.3485
	Hispanic	-25.43 (-101.12, 50.27)	0.5102	18.63 (-54.84, 92.11)	0.6190
	Native American	-97.46 (-222.46, 27.55)	0.1265	-8.58 (-129.7, 112.55)	0.8896
	Other/Asian/ Pacific Islander	119.75 (18.24, 221.26)	0.0208	120.6 (24.19, 217.02)	0.0142
Pre-transplant					
	BMI (kg/m^2^)	-3.42 (-7.25, 0.42)	0.0807	-3.09 (-6.86, 0.68)	0.1086
	Diabetes	-36.11 (-84.7, 12.48)	0.1452	-35.94 (-85.5, 13.63)	0.1552
	Dialysis	-60.29 (-107.86, -12.73)	0.0130	-51.42 (-99.42, -3.42)	0.0358
	CMV seronegative	-31.58 (-76.98, 13.81)	0.1726	29.77 (-15.45, 74.99)	0.1969
Transplant					
	Deceased donor	45.44 (1.01, 89.87)	0.0450	36.2 (-9.47, 81.87)	0.1202
	Lymphocyte depleting agent (for induction or for treating rejection)	-483.22 (-534.95, -431.48)	0.0000	-572.84 (-628.43, -517.25)	0.0000
	Prior kidney transplant	46.35 (-28.69, 121.39)	0.2260	112.86 (41.9, 183.83)	0.0018
	Lymphocyte count at baseline	0.33 (0.3, 0.37)	0.0000	0.35 (0.31, 0.38)	0.0000

A negative estimate refers to a negative correlation between the variable and lymphocyte count.

These correlations are expressed as slopes to account for continuous variables. For example, according to multivariate model, each increase in recipient age by a year resulted in 4.09 (5.88, 2.30, p=0.0001) *fewer* lymphocytes/µl. Thus, a 65-year-old would on average have approximately 123 fewer lymphocytes/µl than a 35-year-old when controlling for other variables. The use of alemtuzumab or rATG at any point in the first 3 years resulted in a mean decrease of 572.84 (628.43, 517.25) lymphocytes/µl (p<0.0001) at 3 years after KTx. Interestingly in a univariate model, as the age of the recipient increases, so does the 3 yr lymphocyte count. However, this is due to the use of basiliximab in older recipients. Once the use of rATG or alemtuzumab induction is added to the model, the age of recipient has a negative correlation with the 3-year lymphocyte count.

### Examining lymphocyte count cutoffs and outcomes

**Figure 2d** above shows that decreasing lymphocyte counts at 3 years after KTx are associated with increasing mortality. To investigate a level that might be employed clinically, we examined multiple lymphocyte cutoff levels from 400 to 3200 lymphocytes/µl (**Table 4**). A lymphocyte count <950 (i.e. the lower level of normal) was the cutoff for significance (HR=1.18, p=0.011, n= 1275, 31% of recipients). Levels below this had increasing risk but had fewer recipients. For example, a lymphocyte count <600 at 3 years had a HR=1.65 (1.35, 2.01, p<0.001) but included only 361/4080 (8.8% of recipients).

**Table 4:** Hazard ratio of DWFG when compared to multiple 3-year lymphocyte values.

Threshold	Number of patients under threshold	HR^a^	P-value
400	98	1.85 (1.31, 2.6)	0.0004
450	153	1.74 (1.31, 2.31)	0.0001
500	205	1.72 (1.34, 2.21)	0
550	294	1.64 (1.32, 2.03)	0
600	361	1.65 (1.35, 2.01)	0
650	477	1.48 (1.24, 1.77)	0
700	581	1.46 (1.24, 1.72)	0
750	735	1.42 (1.22, 1.65)	0
800	829	1.35 (1.17, 1.57)	0
850	986	1.28 (1.12, 1.47)	0.0004
900	1109	1.18 (1.03, 1.35)	0.016
950	1275	1.18 (1.04, 1.35)	0.0111
1000	1416	1.15 (1.01, 1.3)	0.0343
1050	1587	1.11 (0.98, 1.26)	0.0943
1100	1709	1.08 (0.96, 1.23)	0.2028
1150	1865	1.08 (0.95, 1.22)	0.2355
1200	1991	1.06 (0.93, 1.2)	0.3756
1250	2154	1.03 (0.91, 1.16)	0.6489
1300	2283	1.03 (0.91, 1.17)	0.6541
1350	2425	1 (0.88, 1.13)	0.9476
1400	2519	0.97 (0.86, 1.11)	0.6779

a Hazard Ratios relative to the patients above the threshold.

Risk factors for having a lymphocyte count <950/µl at 3 years were similar to those identified when we used lymphocyte counts as a continuous variable (**Supplementary Table S1**). Importantly, of the 1275 recipients with a lymphocyte count <950/µl at 3 years, 1121 (87.9%) received rATG or alemtuzumab for induction, 14 had Simulect induction but received rATG for the treatment of rejection and 140 never received rATG or alemtuzumab. Recipients with very low lymphocyte counts commonly developed lymphopenia early and remained lymphopenic. For example, in recipients with a lymphocyte count <750 at 3 years, the percentage with a lymphocyte count below the normal range at baseline was 26.5%, at 1 year 89.7%, at 2 years, 88.9% and at 4 years 83.4%.

### Recipient age, rATG vs alemtuzumab and mortality

Most recipients ≥ 65 years of age at transplant never received rATG or alemtuzumab. Thus, the mortality risks related to these two agents primarily applies to recipients <65 years of age in our cohort. Of the 1826 recipients who were ≥ 65 years of age at the time of transplantation, only 481 (26.3%) received rATG or alemtuzumab either for induction or for the treatment of rejection in the first 3 years. In those <65 years, the use of T cell depletion was 5048 (92.1%). The demographics of the recipients who received rATG or alemtuzumab is presented in **Supplementary Table S2**. The mean age was lower than that of the overall cohort (49.6 ± 12.6 years). Risk factors for lymphopenia at 3 years (**Table 5**) and mortality (**Table 6**) were similar to the overall cohort. Importantly, compared to rATG, the use of alemtuzumab increased the risk of lymphopenia, but not of mortality.

**Table 5:** Risk factor for lymphopenia in recipients receiving lymphocyte depleting agents.

Category	Variable	Univariable		Multivariable	
		Estimate (cells/ul per value of predictor)	P	Estimate (cells/µl per value of predictor)	P
Demographics					
	Age (years)	-7.04 (-8.86, -5.22)	0.0000	-4.49 (-6.41, -2.57)	0.0000
	Male	-15.37 (-61.81, 31.08)	0.5166	31.18 (-14.27, 76.62)	0.1787
	Race				
	Black	123.08 (58.46, 187.71)	0.0002	87.69 (19.88, 155.51)	0.0113
	Hispanic	63.42 (-12.01, 138.85)	0.0993	73.47 (-3.7, 150.64)	0.062
	Native American	29.18 (-94.57, 152.94)	0.6439	59.80 (-67.11, 186.71)	0.3556
	Other/Asian/ Pacific Islander	256.29 (152.15, 360.43)	<0.001	240.57 (137.26, 343.87)	<0.001
Pre-transplant					
	BMI (kg/m^2^)	-3.50 (-7.36, 0.36)	0.0754	-4.15 (-8.09, -0.21)	0.0388
	Diabetes	-45.56 (-97.05, 5.92)	0.0828	-14.18 (-68.42, 40.06)	0.6083
	Dialysis	38.22 (-12.16, 88.59)	0.137	-23.37 (-76.11, 29.38)	0.3851
	CMV seropositive	23.37 (23.73, 70.46)	0.3307	-43.93 (-92.33, 4.47)	0.0752
Transplant					
	Deceased donor	68.12 (22.11, 114.14)	0.0037	43.97 (-5.47, 93.41)	0.0813
	Campath (alemtuzumab)	-62.4 (-108.54, -16.25)	0.0081	-72.51 (-119.04, -25.98)	0.0023
	Prior kidney transplant	146.45 (75.12, 217.78)	0.0001	101.59 (28.95, 174.24)	0.0061
	Lymphocyte count at baseline	0.28 (0.24, 0.31)	<0.001	0.27 (0.24, 0.31)	<0.001

**Table 6:** Risk factors for mortality in recipients receiving rATG or alemtuzumab.

Category	Variable	Univariable		Multivariable	
		HR (95% CI)	P	HR (95% CI)	P
Demographics					
	Age (years)	1.06 (1.06, 1.07)	<0.001	1.06 (1.06, 1.07)	<0.001
	Male	1.17 (1.05, 1.31)	0.0045	1.09 (0.93, 1.29)	0.2916
	Race				
	Black	1.16 (0.98, 1.36)	0.0802	0.78 (0.61, 1.00)	0.0481
	Hispanic	1.17 (0.99, 1.38)	0.073	0.97 (0.73, 1.28)	0.8132
	Native American	1.81 (1.43, 2.28)	<0.001	1.17 (0.78, 1.76)	0.4491
	Other/Asian/ Pacific Islander	0.62 (0.45, 0.85)	0.0032	0.51 (0.32, 0.79)	0.0031
Pre-transplant					
	BMI (kg/m^2^)	1.03 (1.02, 1.03)	<0.001	1.00 (0.99, 1.01)	0.968
	Diabetes	2.99 (2.68, 3.34)	<0.001	2.19 (1.84, 2.61)	<0.001
	Dialysis	1.91 (1.67, 2.19)	<0.001	1.61 (1.32, 1.96)	<0.001
	CMV seropositive	1.24 (1.11, 1.39)	0.0002	1.08 (0.91, 1.29)	0.3778
Transplant					
	Deceased donor	2.04 (1.83, 2.29)	<0.001	1.56 (1.31, 1.85)	<0.001
	Campath (alemtuzumab)	0.99 (0.89, 1.11)	0.9138	1.08 (0.91, 1.29)	0.3566
	Prior kidney transplant	0.73 (0.61, 0.88)	0.0008	1.34 (1.02, 1.76)	0.0361
	Lymphocyte count at baseline < 950	1.16 (1.00, 1.35)	0.0488	1.03 (0.83, 1.27)	0.8051
	Lymphocyte count at 3 years < 950	1.51 (1.29, 1.77)	<0.001	1.27 (1.08, 1.5)	0.0045

### Impact of lymphocyte count at 3 years on graft loss

Finally, we examined the impact of clinical factors including lymphocyte counts at 3 years on graft loss and found that a lymphocyte count <950 was associated with an increased rate of graft loss (HR 1.70, p<0.001). Other factors were deceased donor recipient (HR 1.38, p<0.0016), Black or Native American recipient (compared to White/non-Hispanic) (HR 1.52, p<0.001 and HR 1.61, p<0.0297, respectively), pretransplant dialysis (HR 1.52, p<0.001), and CMV positive serologic status at the time of transplant (HR 1.23, p<0.0419).

### Phenotypic analysis of peripheral blood lymphocytes

#### Baseline characteristics of the cohort

A subset of 84 recipients underwent immunotyping via CyTOF three years post-KTx. This cohort was selected to represent a diverse range of lymphocyte counts, CMV serostatus, age, and death with a functioning graft (DWFG). The demographic characteristics are summarized in **Table 7**. Notably, 63.1% of the cohort had lymphocyte counts below 950, 45.2% were CMV seropositive at the time of transplant, 63.1% required pretransplant dialysis, and 77.4% received either rATG or alemtuzumab.

**Table 7:** Baseline characteristics of the CyTOF cohort.

Age in years (median [IQR])	Overall (N=84)
	56 [47, 63]
Sex (%)	
Female	35 (41.7)
Male	49 (58.3)
BMI (median [IQR])	28.4 [25.5, 32.7]
Race (%)	
Caucasian	63 (75.0)
Black	10 (11.9)
Hispanic	7 (8.3)
Native American	2 (2.4)
Other	2 (2.4)
CMV status (%)	
Negative	46 (54.8)
Positive	38 (45.2)
Pretransplant dialysis (%)	
Yes	53 (63.1)
No	31 (36.9)
Pretransplant diabetes (%)	
Type 2	10 (11.9)
Type 1	7 (8.3)
No	67 (79.8)
Induction type	
Thymoglobulin	33 (39.3)
Alemtuzumab	32 (38.1)
Simulect (Basiliximab)	14 (16.7)
Other	5 (6.0)
Graft type (%)	
CAD	39 (46.4)
LRD	15 (17.9)
LURD	30 (35.7)
ALC at 3y (median [IQR])	0.63 [0.57, 1.20]
ALC at 3y <950/uL (%)	53 (63.1)

#### Immune cell phenotypes and lymphocyte count

CyTOF immune phenotyping was conducted on PBMCs collected three years post-KTx from cohort recipients using the biomarkers detailed in **Supplementary Table S3**. The definition of cell subsets, based on gating strategies, is summarized in **Supplementary Table S4**, while a categorization algorithm specific for T cell subsets is provided in **Supplementary Figure S1**.

Analysis of lymphocyte subsets showed a correlation between decreasing lymphocyte counts and reduced absolute numbers of total B, NK, and T cells (**Supplementary Figure S2**). This included both CD4+ and CD8+ T cells, as well as all defined T cell subsets (**Supplementary Figure S2**). The correlations were quite high (r ≥ 0.65) for T cells, B cells, NK cells, CD4+ T cells, CD8 T cells and CD4 transitional memory cells. In general, cell types that were more common even with lymphopenia showed lower variability and thus higher correlations. Importantly, cell subsets crucial for immune reconstitution, such as recent thymic emigrants (CD31+), naïve T cells (CD45RO-, CCR7+. CD28+, CD95-) and stem cell-like memory T cells (T_SCM_, CD45RO-, CCR7+. CD28+, CD95+) were all diminished in recipients with lymphopenia three years after KTx (**Supplementary Figure S2**). FoxP3+ T cells (putative regulatory T cells) decreased with lymphopenia as did CD8+ T cells (data not shown). The relative proportions of each of these lymphocyte subsets were similar with different lymphocyte counts suggesting a non-selective reduction across all populations (**Supplementary Figure S3**).

To investigate the mechanisms underlying the observed decrement, we examined the expression of markers indicative of senescence (CD57), exhaustion (KLRG-1, PD-1, GZMK), and recent proliferation (Ki-67). The proportions of cells expressing these markers were largely invariant across a range of absolute lymphocyte counts. However, a significant inverse correlation was identified between absolute lymphocyte count and the frequency of PD-1+ T and B cells, suggesting an enrichment of exhausted T and B cells in individuals with lymphopenia (**Supplementary Figure S4G, H**). The expression of KLRG-1 and GZMK was not increased with lymphopenia. Finally, the expression of PD-1 was increased with lymphopenia in CD4+ and CD8+ T cells and in those subsets related to homeostasis: CD4+ naïve T cells, CD4 recent thymic immigrants, CD4 T_SCM_ and CD8 naïve T cells (**Supplementary Figure S5**) Importantly, CD4+ T_SCM_ also had increased CD57 expression suggesting an increased proportion of senescent cells with lymphopenia (**Supplementary Figure S5**, p= 0.042, r=- 0.22).

## Discussion

This large, multicenter, retrospective study of 7307 solitary kidney transplants, found that persistent lymphopenia (a lymphocyte count below the normal range, <950μl) was common after kidney transplantation while leukopenia was rare. The prevalence peaked at 54% at 1 year and then stabilized around 30% at 3 years and beyond. Early lymphopenia was not associated with mortality, but at 3 years and beyond, a progressively lower lymphocyte count correlated with an increasing risk of mortality. In a multivariable model, risk factors for a lower lymphocyte count were the use of rATG or alemtuzumab either for induction or for the treatment of rejection, increasing recipient age, pretransplant dialysis, a prior kidney transplant and a lower lymphocyte count pretransplant.

Relatively few studies have examined persistent lymphopenia after KTx in detail. An early study from 2010 (3) studied CD4 T cell numbers in 301 KTx recipients treated with ATG, cyclosporine and azathioprine with a mean follow up of 7.66 years. Similar to our study, they found that at 2 years after KTx, 27% had CD4 T cell lymphopenia (< 300 cells/ul) and that this cutoff increased mortality (24.1% vs 7.6%, HR 4.63). They correlated CD4 reconstitution with thymic function (using T cell receptor excision circles) and showed that a higher number of recent thymic emigrants (i.e. higher levels of excision circles) correlated with lower risk for cancer and infection. In the same cohort, the group also noted that prolonged lymphopenia related to atherosclerosis in a much shorter follow-up period (23.5 months on average) (4).

Our studies both confirm their findings and extend our understanding in several ways. We show in a more contemporary population that lymphopenia at 3 years involves not only CD4+ T cells, but also almost all T cell types, B cells and NK cells. To our knowledge, this is the first to show that lymphopenia years after KTx also involves decreased numbers of B and NK cells. Our data also show that both rATG and alemtuzumab are major driver of persistent lymphopenia with the latter showing a significantly higher risk. We also show that a few recipients who never received these drugs can have persistent lymphopenia and that other factors such as baseline lymphocyte count, pretransplant dialysis, a prior kidney transplant and recipient age also are important.

These data also shed new light on the possible cellular mechanisms of persistent lymphopenia. We believe this is the first study to examine T stem cell memory cells in KTx recipients. T_SCM_ are a rare subset of memory lymphocytes endowed with the stem cell–like ability to self-renew and the multipotent capacity to reconstitute the entire spectrum of memory and effector T cell subsets. Cumulative evidence in mice, nonhuman primates, and humans indicate that T_SCM_ cells are minimally differentiated cells at the apex of the hierarchical system of memory T lymphocytes (5–8). Widespread acceptance of their existence and techniques to identify them in cohorts like ours have only occurred in the past decade. Some controversy exists over the terminology with one group suggesting the term “precursors exhausted” T cells (9) to contrast them with the terminal exhausted effector T cells. Our data show that T_SCM_ are decreased in recipients with lymphopenia and that this also correlates with a decrease in all types of memory T cells and terminally-differentiated effector cells. Other cell types involved in maintaining normal levels of T cells were also affected. Decreased thymic activity and decreased numbers of naïve CD4+ and CD8+ T cells also correlate with lymphopenia.

An increase in PD-1+ expression (a marker of exhaustion) correlated with decreasing numbers of NK cells and a wide variety of T cells including CD4+ naïve, CD4+ recent thymic emigrants, CD4 T_SCM_ and CD8+ naïve T cells. In addition, CD4+ T_SCM_ also showed increased proportions of CD57+ (senescent) T cells. T cell exhaustion and senescence have been described previously in KTx recipients but have not been linked to lymphopenia. Almost all prior studies of peripheral blood T cells have only examined the proportion of the cell types (and did not include the total number). In addition, most focused on early time points and did not show correlations with mortality. Luttropp et al. (10) showed that telomere length (a correlate to CD57+ expression) was significantly decreased at 1 year after transplantation. Fribourg et al. (11) showed that the proportion of CD4 and CD8+ exhausted T cells increased after by 6 months after KTx and was more profound in recipients treated with ATG. Schaenman et al. (12) found that at 4–6 months after KTx, older recipients had increased proportions of exhausted (KLRG-1) and senescent (CD57+) CD4 and CD8+ T cells. In our cohort, recipients with normal range lymphocyte counts at 3 years appeared to have fewer exhausted and senescent cells than in these prior studies. We hypothesize that this is because our studies examined the immune system at a time point when immune reconstitution has likely reached its peak in most recipients and that the percentage of exhausted and senescent T cells had decreased. Our data suggest that mortality is related to the persistence of lymphopenia in a small subset of recipients rather than lymphopenia at early time points.

Taken together, these data are consistent with a hypothesis that persistent lymphopenia is at least partially due to a decrease in the normal homeostatic mechanisms that maintain the various T cells. Other potential mechanisms not studied here include impairment in the bone marrow production of precursors and increased destruction of lymphocytes peripherally. The fact that B cells and NK cells also are decreased suggests that these latter mechanisms may be important and deserve further study.

Limitations of our study are the fact that it is retrospective. While the patient population was diverse, it does not completely recapitulate the entire US transplant population. Our protocol generally avoided rATG and alemtuzumab in older recipients and thus limited our assessment of lymphopenia in this important population. Studies of these agents in this older age group would be important. Although we performed an extensive review for the use of T-cell depleting agents prior to 3 years post-transplant, assessing changes in maintenance immunosuppression was not possible at the scale of the population which may confound results. Our recent study of a subset of our cohort suggested that almost half of the recipients were receiving 1 g MMF per day or less at 1 and 5 years after KTx (13).

Additionally, the immune system does not work in isolation. Studies have found that cytokines involved in chronic inflammation, termed “inflammaging”, or senescent-associated secretory proteins such as IL-6 and GDF-15, have been implicated with decreased survival in normal aging, disease states like end stage renal disease, and in KTx recipients (14–20). While we focused on the association between lymphocyte count and immunophenotypes and the risk for DWFG in our study, the relationship between immune cell types and cytokines and their impact on survival and vaccine response are also critical area for additional studies. Finally, we note that the relationships herein a correlations rather than causations. For example, a patient who died due to a malignancy may have had a low ALC value due to that malignancy rather than the other way around.

The cut-off of 950 cells/ul was chosen not just because it reflected a change point in the relationship between ALC and mortality, but it is also the bottom of the most recent reference range for our institution and similar to published literature (21). When interpreting these results note that this may be context specific: For example, in the Common Terminology Criteria for Adverse Events (CTCAE) two grades of lymphopenia severity exist below this threshold (<200 cells/uL and 200–500 cells/uL).

We conclude that the peripheral blood lymphocyte count is an easily measured and important biomarker to aid in the management of kidney transplant recipients. Prospective studies and clinical trials are needed to determine whether changes in immunosuppression or other aspects of management can maintain normal lymphocyte counts and improve outcomes.

## Supplementary methods

CMV prophylaxis and induction therapy selection:

The current clinical guideline for cytomegalovirus (CMV) prophylaxis for seronegative recipients who received kidneys from seropositive donors (i.e. CMV R-/D+) at Mayo Clinic is administration of oral valganciclovir 900mg daily for 6months or ganciclovir 5mg/kg IV daily for 6months for recipients who are unable to tolerate enteral medications. After the 6-month period, recipients who are at high risk for developing severe CMV disease will be monitored via weekly CMV PCR testing. Those who are not considered at high risk will undergo routine follow-up clinical visit and CMV PCR testing performed for cause. CMV viremia is defined as detectable levels of CMV on PCR testing while CMV infection is defined as clinical presentation of signs and symptoms associated with CMV as determined by physicians.

For induction therapy selection, most transplant recipients will receive either standard dosage of alemtuzumab or thymoglobulin following transplantation. However, in our clinical practice, recipients who are aged 65 or older or considered to be high risk for development of infection or post-transplant lymphoproliferative disorder are given basiliximab for induction therapy. Following induction, recipients are discharged with standard maintenance immunosuppressive regimen consisting of calcineurin inhibitor, mycophenolate mofetil, and steroid.

*Frozen peripheral blood mononuclear cells (PBMC)*

In addition to routine standard of care laboratory evaluation, peripheral blood was obtained from recipients at time of transplant and at all subsequent routine visits. These peripheral blood samples were processed using standard Ficoll gradient separation at room temperature and the resulting mononuclear cell layer were collected, frozen, and stored in a biobank repository.

Cytometry by Time of Flight (CyTOF)

A random cohort of recipients with at least three years of post-KTx follow up and frozen PBMC samples were selected to undergo immunophenotyping via CyTOF.

*Reagents*

The Maxpar^®^ Direct™ Immune Profiling Assay™ (PN 201325) was used to stain and prepare PBMC for CyTOF. This kit includes Cell Staining Buffer (CSB), Fix and Perm Buffer, PBS, antibodies lyophilized in 5 mL tube (**Table 1**) and Cell-ID Intercalator-Ir. Cell-ID™ Cisplatin-195Pt (PN 201195), Perm-S solution (201066) and EQ Four Element Calibration Beads (PN 201078) were purchased from Standard BioTools. Human TruStain FcX™ (Fc receptor blocking solution) was purchased from Biolegend (422302). The FoxP3/Transciption Factor Staining Buffer set was purchased from ThermoFisher Scientific. Paraformaldehyde (PFA; 15710) was purchased from EM Sciences and 10X PBS pH 7.2 (MB-008) was purchased from Rockland. The pre-conjugated antibody to PD-1 was purchased from Standard BioTools. The antibodies CD62L, CD95, CD31, Tim-3 and KLRG-1 were purchased from Biolegend. Granzyme-K antibody was purchased from Santa Cruz. Unconjugated antibodies were purchased were custom conjugated in-house through the Mayo Clinic Hybridoma Core using Maxpar X8 or MCP9 antibody labeling kits (Standard BioTools) (see **Supplementary Tables S1**, **S2** for complete markers and associated phenotypes).

*Samples and Processing*

Peripheral blood mononuclear cells processed and recovered from cryopreservation on the day of preparation for CyTOF. One to 3 million cells were suspended in 1 mL of CSB after recovery from cryopreservation. Each sample was incubated for 5 minutes with a 0.5 um Cisplatin-195Pt solution in PBS. Samples were then centrifuged and washed twice with CSB. After the addition of Fc receptor block, cells were added to a 5 mL tube containing lyophilized antibodies. Upon reconstitution of the pellet additional antibodies in solution were added to the cells. Samples were then incubated at room temperature for 30 minutes. After washing twice with CSB, samples were fixed with 2% PFA in PBS. After fixation and wash, cells were permeabilized using FoxP3/Transcription staining buffer (Thermo-Fisher) and then resuspended in permeabilization solution containing antibodies to intracellular and nuclear markers before incubation at room temperature for 45 minutes. Cells were washed twice with CSB and then resuspended in 30 nM intercalation solution and incubated at 4 °C on a rocker overnight. To facilitate batch variation a replicate reference PBMC sample derived from apheresis cones prepared with samples on each staining day. Samples were prepared in batches of 7 to 8 samples. After overnight incubation with intercalation solution samples were washed and resuspended in cell acquisition solution containing a 1:10 dilution of EQ four element calibration beads. Prior to data acquisition samples were filtered through a 35 um blue cap tube (Falcon; 35209).

*Mass Cytometry and Data Acquisition*

Samples were loaded onto a Helios CyTOF^®^ system (Standard BioTools) using an attached autosampler and were acquired at a rate of 200–400 events per second. Data were collected as.FCS files using the CyTOF software and after acquisition intra file signal drift was normalized to the acquired calibration bead signal using the normalization algorithm embedded in the CyTOF software (version 7.05189.0; Standard BioTools).

*CyTOF data analysis*

FCS files were demultiplexed and normalized to account for drift in marker expression over time. Marker distributions between batches were normalized using the R package CytoNorm (https://github.com/saeyslab/CytoNorm), training the model on technical replicates of a reference sample run in each batch. For each marker and for each sample, the threshold for positive/negative marker expression was calculated by summarizing expression as histogram bins and using algorithm to identify peaks and valleys. The first valley from negative expression represented the threshold between positive (denoted +) and negatively (denoted -) expressing cells. Removal of multiplets, dead cells, and debris was performed by selecting for Beads-, Pt195-, and the largest population of the Bayesian metrics (Offset, Width, Event Length) and DNA1/DNA2.

To visualize the gated cell populations, samples were down-sampled to a uniform 20,000 cells per sample. Cell phenotypes were determined based on the marker panel detailed in **Supplementary Table S3**, with T cell subset identification following the algorithm outlined in **Supplementary Figure S1**. Absolute cell counts were derived by applying the proportional representation of each phenotype within the total lymphocyte count (defined as the sum of T, B, and NK cells) to the recipient’s lymphocyte count at 3 years post-transplantation. Phenotypic proportions are expressed as percentages relative to their respective parental cell populations (T, B, NK, CD4+ T, or CD8+ T cells).

## Data Availability

The data set contains potentially identifiable information such as transplant and lab dates which can not be made public due to institution IRB restrictions. A minimal, de-identified dataset may be provided upon reasonable request by contacting the corresponding author.

## Supplementary material

Supplementary Figure 1 Categorization algorithm for T cell subsets.

Supplementary Figure 2 Scatterplots showing the correlation of lymphocyte counts (x10^6^ cells/ml) with absolute counts (x10^6^ cells/ml) of each studied immune cell phenotypes.

Supplementary Figure 3 Scatterplots showing the correlation of lymphocyte counts (x10^6^ cells/ml) with proportion of selected CD4+ and CD8+ T cell subsets.

Supplementary Figure 4 Scatterplots showing the correlation of lymphocyte counts (x10^6^ cells/ml) with proportions of T, B and NK cells expressing functional markers CD57, KLRG-1, PD-1, GZMK, Ki-67.

Supplementary Figure 5 Scatterplots showing significant correlations of lymphocyte counts (x10^6^ cells/ml) with proportions of CD4+ and CD8+ T cell subsets expressing functional markers.

Supplementary Table 1 Risk factors for mortality (<950 cohort at 3 years).

Supplementary Table 2 The demographics of the recipients who received rATG or alemtuzumab.

Supplementary Table 3 CyTOF immune profiling panel consisting of 16 biomarkers.

Supplementary Table 4 Gating strategies for cell subset definition.
