# Supplementary material for: Persistent lymphopenia after kidney transplantation: increased mortality and decreased homeostatic mechanisms
Source: Front Immunol. 2025 Jun 19;16:1605794. doi: 10.3389/fimmu.2025.1605794 (PMC12221922; doi:10.3389/fimmu.2025.1605794)
Supplement: Supplementary Figure 1 — Categorization algorithm for T cell subsets. [file DataSheet1.docx]

**Supplemental Table 1:** Risk factors for mortality (<950 cohort at 3 years).

|  |  | Univariable | | Multivariable | |
| --- | --- | --- | --- | --- | --- |
| Category | Variable | HR (95% CI) | P | HR (95% CI) | P |
| Demographics | |  |  |  |  |
|  | Age (years) | 1.07 (1.06, 1.07) | <0.001 | 1.07 (1.06, 1.07) | <0.001 |
|  | Male | 1.27 (1.16, 1.4) | <0.001 | 1.12 (0.98, 1.28) | 0.106 |
|  | Race |  |  |  |  |
|  | Black | 1.05 (0.92, 1.21) | 0.4766 | 0.77 (0.62, 0.95) | 0.014 |
|  | Hispanic | 1.04 (0.9, 1.2) | 0.6288 | 1.01 (0.8, 1.28) | 0.9418 |
|  | Native American | 1.52 (1.24, 1.88) | 0.0001 | 1.32 (0.94, 1.87) | 0.112 |
|  | Other/Asian/  Pacific Islander | 0.71 (0.56, 0.9) | 0.0046 | 0.49 (0.34, 0.71) | 0.0002 |
| Pre-transplant | |  |  |  |  |
|  | BMI (kg/m^2^) | 1.02 (1.01, 1.03) | <0.001 | 1 (0.99, 1.01) | 0.941 |
|  | Diabetes | 2.8 (2.56, 3.05) | <0.001 | 2.06 (1.79, 2.37) | <0.001 |
|  | Dialysis | 1.72 (1.55, 1.91) | <0.001 | 1.61 (1.38, 1.88) | <0.001 |
|  | CMV seropositive | 0.83 (0.76, 0.91) | <0.001 | 0.91 (0.78, 1.04) | 0.1705 |
| Transplant | |  |  |  |  |
|  | Deceased donor | 2 (1.83, 2.19) | <0.001 | 1.53 (1.33, 1.76) | <0.001 |
|  | Lymphocyte depleting agent | 0.43 (0.39, 0.47) | <0.001 | 0.79 (0.67, 0.92) | 0.0037 |
|  | Prior kidney transplant | 0.65 (0.55, 0.76) | <0.001 | 1.35 (1.06, 1.73) | 0.0169 |
|  | Lymphocyte count at baseline < 950 | 1.09 (0.97, 1.23) | 0.1665 | 0.92 (0.77, 1.1) | 0.3686 |
|  | Lymphocyte count at 3 years < 950 | 1.18 (1.04, 1.35) | 0.0111 | 1.33 (1.15, 1.53) | 0.0001 |

**Supplemental Table 2:** The demographics of the recipients who received rATG or alemtuzumab.

| Category | Variable | Overall (N = 4080) | ALC >= 950 | ALC < 950 | p-value |
| --- | --- | --- | --- | --- | --- |
| Demographics | Age | 53.816 (14.532) | 53.532 (12.187) | 53.727 (13.841) | 0.543 |
|  | Age >55 | 1482 (52.8%) | 681 (53.4%) | 2163 (53.0%) | 0.732 |
|  | Male | 1635 (58.3%) | 747 (58.6%) | 2382 (58.4%) | 0.857 |
|  | Race/Ethnicity |  |  |  | 0.002 |
|  | Caucasian/Non-Hispanic | 2769 (67.9%) | 1867 (66.6%) | 902 (70.7%) |  |
|  | Black | 564 (13.8%) | 390 (13.9%) | 174 (13.6%) |  |
|  | Hispanic | 402 (9.9%) | 283 (10.1%) | 119 (9.3%) |  |
|  | Native American | 135 (3.3%) | 95 (3.4%) | 40 (3.1%) |  |
|  | Other | 210 (5.1%) | 170 (6.1%) | 40 (3.1%) |  |
| Pre-transplant |  |  |  |  |  |
|  | BMI | 28.604 (5.710) | 28.723 (5.973) | 28.641 (5.793) | 0.543 |
|  | Dialysis | 2771 (67.9%) | 1875 (66.8%) | 896 (70.3%) | 0.030 |
|  | Diabetes | 1215 (29.8%) | 826 (29.5%) | 389 (30.5%) | 0.500 |
|  | CMV seronegative | 1621 (39.7%) | 1027 (36.6%) | 594 (46.6%) | <0.001 |
| Transplant |  |  |  |  |  |
|  | Deceased donor | 1992 (48.8%) | 1381 (49.2%) | 611 (47.9%) | 0.437 |
|  | Induction |  |  |  |  |
|  | Lymphocyte depleting |  |  |  | <0.001 |
|  | Campath (Alemtuzumab) | 1627 (39.9%) | 1014 (36.2%) | 613 (48.1%) |  |
|  | Thymoglobulin (rATG) | 1411 (34.6%) | 903 (32.2%) | 508 (39.8%) |  |
|  | Non depleting |  |  |  |  |
|  | Other | 48 (1.2%) | 30 (1.1%) | 18 (1.4%) |  |
|  | Simulect (Basiliximab) | 992 (24.3%) | 856 (30.5%) | 136 (10.7%) |  |
|  | None | 48 (1.2%) | 30 (1.1%) | 18 (1.4%) |  |
|  | Prior kidney transplant | 396 (9.7%) | 285 (10.2%) | 111 (8.7%) | 0.146 |
|  | 3 year white blood cell count (x10^6^ cells/ml) | 6.644 (2.500) | 7.018 (2.355) | 5.822 (2.610) | <0.001 |
|  | 3 year absolute lymphocyte count (x10^6^ cells/ml) | 1346.360 (723.800) | 1648.177 (674.374) | 682.362 (185.991) | <0.001 |
|  | Rejection in the last 3 years | 881 (26.6%) | 573 (25.2%) | 308 (29.7%) | 0.007 |
|  | Follow-up time (years) | 8.280 (3.924) | 8.357 (3.915) | 8.109 (3.939) | 0.061 |
|  | Graft status |  |  |  | <0.001 |
|  | Active | 2558 (62.7%) | 1827 (65.1%) | 731 (57.3%) |  |
|  | Death with Function | 1008 (24.7%) | 667 (23.8%) | 341 (26.7%) |  |
|  | Graft loss | 514 (12.6%) | 311 (11.1%) | 203 (15.9%) |  |
|  | Causes of Death |  |  |  | 0.852 |
|  | Cancer | 124 (14.3%) | 84 (14.6%) | 40 (13.7%) |  |
|  | Cardiac | 81 (9.3%) | 54 (9.4%) | 27 (9.2%) |  |
|  | Infection | 188 (21.6%) | 130 (22.5%) | 58 (19.8%) |  |
|  | Other | 85 (9.8%) | 56 (9.7%) | 29 (9.9%) |  |
|  | Unknown | 392 (45.1%) | 253 (43.8%) | 139 (47.4%) |  |

**Supplemental Table 3:** CyTOF Immune Profiling Panel Consisting of 16 Biomarkers.

| **Marker** | **Clone** | **Metal Label** | **Localization** | **Company** | **Panel** | **Catalog No.** |
| --- | --- | --- | --- | --- | --- | --- |
| CD45 | HI30 | 089Y | Surface | Standard BioTools | Maxpar Direct IPA | 201334 |
| CD3 | UCHT1 | 170Er | Surface | Standard BioTools | Maxpar Direct IPA | 201334 |
| CD19 | HIB19 | 144Nd | Surface | Standard BioTools | Maxpar Direct IPA | 201334 |
| CD56 | NCAM16.2 | 163Dy | Surface | Standard BioTools | Maxpar Direct IPA | 201334 |
| CD4 | RPA-T4 | 145Nd | Surface | Standard BioTools | Maxpar Direct IPA | 201334 |
| CD8a | RPA-T8 | 146Nd | Surface | Standard BioTools | Maxpar Direct IPA | 201334 |
| CD45RO | UCHL1 | 149Sm | Surface | Standard BioTools | Maxpar Direct IPA | 201334 |
| CCR7 | G043H7 | 167Er | Surface | Standard BioTools | Maxpar Direct IPA | 201334 |
| CD28 | CD28.2 | 160Gd | Surface | Standard BioTools | Maxpar Direct IPA | 201334 |
| CD95 | DX2 | 116Cd | Surface | Biolegend | Manual Addition | 306631 |
| CD31 | WM59 | 142Nd | Surface | Biolegend | Manual Addition | 303127 |
| FoxP3 | 259D/47 | 113Cd | Intracellular | Biolegend | Manual Addition | 320202 |
| CD57 | HCD57 | 155Gd | Surface | Standard BioTools | Maxpar Direct IPA | 201334 |
| KLRG-1 | SA231A2 | 165Ho | Intracellular | Biolegend | Manual Addition | 367702 |
| PD-1 | EH12.2H7 | 175Lu | Surface | Standard BioTools | Manual Addition | 31750158 |
| GZMK | GMBC3 | 169Tm | Intracellular | Santa Cruz | Manual Addition | sc-56125 |
| Ki-67 | Ki-67 | 106Cd | Nuclear | Biolegend | Manual Addition | 350523 |

**Supplemental Table 4:** Gating Strategies for Cell Subset Definition.

| **Phenotype** | **Cell Markers** |
| --- | --- |
| Leukocytes | CD45+ |
| T cells | CD45+CD3+ |
| B cells | CD45+CD19+ |
| NK cells | CD45+CD56+ |
| CD4 T cells | CD45+CD3+CD4+ |
| CD8 T cells | CD45+CD3+CD8+ |
| Regulatory T cells (TREG) | CD45+CD3+CD4+FOXP3+CD25+ |
| CD4 naïve T cells (TN) | CD45+CD3+CD4+CD45RO-CCR7+CD28+CD95- |
| CD4 stem cell memory (T_SCM_) | CD45+CD3+CD4+CD45RO-CCR7+CD28+CD95+ |
| CD4 central memory (TCM) | CD45+CD3+CD4+CD45RO+CCR7+CD28+ |
| CD4 transitional memory (TTM) | CD45+CD3+CD4+CD45RO+CCR7-CD28+ |
| CD4 effector memory (TEM) | CD45+CD3+CD4+CD45RO+CCR7-CD28- |
| CD4 terminal effector (TTE) | CD45+CD3+CD4+CD45RO-CCR7-CD28- |
| CD8 naïve T cells (TN) | CD45+CD3+CD8+CD45RO-CCR7+CD28+CD95- |
| CD8 stem cell memory (T_SCM_) | CD45+CD3+CD8+CD45RO-CCR7+CD28+CD95+ |
| CD8 central memory (TCM) | CD45+CD3+CD8+CD45RO+CCR7+CD28+ |
| CD8 transitional memory (TTM) | CD45+CD3+CD8+CD45RO+CCR7-CD28+ |
| CD8 effector memory (TEM) | CD45+CD3+CD8+CD45RO+CCR7-CD28- |
| CD8 terminal effector (TTE) | CD45+CD3+CD8+CD45RO-CCR7-CD28- |
| CD4 recent thymic emigrants (RTE) | CD45+CD3+CD4+CD45RO-CCR7+CD31+ |
|  |  |
| **ki-67 overlay** |  |
| T cells ki-67 | CD45+CD3+ki-67+ |
| B cells ki-67 | CD45+CD19+ki-67+ |
| NK cells ki-67 | CD45+CD56+ki-67+ |
| CD4 T cells ki-67 | CD45+CD3+CD4+ki-67+ |
| CD8 T cells ki-67 | CD45+CD3+CD8+ki-67+ |
| Regulatory T cells (TREG) ki-67 | CD45+CD3+CD4+FOXP3+CD25+ki-67+ |
| CD4 naïve T cells (TN) ki-67 | CD45+CD3+CD4+CD45RO-CCR7+CD28+CD95-ki-67+ |
| CD4 stem cell memory (T_SCM_) ki-67 | CD45+CD3+CD4+CD45RO-CCR7+CD28+CD95+ki-67+ |
| CD4 central memory (TCM) ki-67 | CD45+CD3+CD4+CD45RO+CCR7+CD28+ki-67+ |
| CD4 transitional memory (TTM) ki-67 | CD45+CD3+CD4+CD45RO+CCR7-CD28+ki-67+ |
| CD4 effector memory (TEM) ki-67 | CD45+CD3+CD4+CD45RO+CCR7-CD28-ki-67+ |
| CD4 terminal effector (TTE) ki-67 | CD45+CD3+CD4+CD45RO-CCR7-CD28-ki-67+ |
| CD8 naïve T cells (TN) ki-67 | CD45+CD3+CD8+CD45RO-CCR7+CD28+CD95-ki-67+ |
| CD8 stem cell memory (T_SCM_) ki-67 | CD45+CD3+CD8+CD45RO-CCR7+CD28+CD95+ki-67+ |
| CD8 central memory (T_SCM_) ki-67 | CD45+CD3+CD8+CD45RO+CCR7+CD28+ki-67+ |
| CD8 transitional memory (TTM) ki-67 | CD45+CD3+CD8+CD45RO+CCR7-CD28+ki-67+ |
| CD8 effector memory (TEM) ki-67 | CD45+CD3+CD8+CD45RO+CCR7-CD28-ki-67+ |
| CD8 terminal effector (TTE) ki-67 | CD45+CD3+CD8+CD45RO-CCR7-CD28-ki-67+ |
| CD4 recent thymic emigrants (RTE) ki-67 | CD45+CD3+CD4+CD45RO-CCR7+CD31+ki-67+ |
|  |  |
| **PD1 overlay** |  |
| T cells PD1 | CD45+CD3+PD1+ |
| B cells PD1 | CD45+CD19+PD1+ |
| NK cells PD1 | CD45+CD56+PD1+ |
| CD4 T cells PD1 | CD45+CD3+CD4+PD1+ |
| CD8 T cells PD1 | CD45+CD3+CD8+PD1+ |
| Regulatory T cells (TREG) PD1 | CD45+CD3+CD4+FOXP3+CD25+PD1+ |
| CD4 naïve T cells (TN) PD1 | CD45+CD3+CD4+CD45RO-CCR7+CD28+CD95-PD1+ |
| CD4 stem cell memory (T_SCM_) PD1 | CD45+CD3+CD4+CD45RO-CCR7+CD28+CD95+PD1+ |
| CD4 central memory (TCM) PD1 | CD45+CD3+CD4+CD45RO+CCR7+CD28+PD1+ |
| CD4 transitional memory (TTM) PD1 | CD45+CD3+CD4+CD45RO+CCR7-CD28+PD1+ |
| CD4 effector memory (TEM) PD1 | CD45+CD3+CD4+CD45RO+CCR7-CD28-PD1+ |
| CD4 terminal effector (TTE) PD1 | CD45+CD3+CD4+CD45RO-CCR7-CD28-PD1+ |
| CD8 naïve T cells (TN) PD1 | CD45+CD3+CD8+CD45RO-CCR7+CD28+CD95-PD1+ |
| CD8 stem cell memory (T_SCM_) PD1 | CD45+CD3+CD8+CD45RO-CCR7+CD28+CD95+PD1+ |
| CD8 central memory (TCM) PD1 | CD45+CD3+CD8+CD45RO+CCR7+CD28+PD1+ |
| CD8 transitional memory (TTM) PD1 | CD45+CD3+CD8+CD45RO+CCR7-CD28+PD1+ |
| CD8 effector memory (TEM) PD1 | CD45+CD3+CD8+CD45RO+CCR7-CD28-PD1+ |
| CD8 terminal effector (TTE) PD1 | CD45+CD3+CD8+CD45RO-CCR7-CD28-PD1+ |
| CD4 recent thymic emigrants (RTE) PD1 | CD45+CD3+CD4+CD45RO-CCR7+CD31+PD1+ |
|  |  |
| **CD57 overlay** |  |
| T cells CD57 | CD45+CD3+CD57+ |
| B cells CD57 | CD45+CD19+CD57+ |
| NK cells CD57 | CD45+CD56+CD57+ |
| CD4 T cells CD57 | CD45+CD3+CD4+CD57+ |
| CD8 T cells CD57 | CD45+CD3+CD8+CD57+ |
| Regulatory T cells (TREG) CD57 | CD45+CD3+CD4+FOXP3+CD25+CD57+ |
| CD4 naïve T cells (TN) CD57 | CD45+CD3+CD4+CD45RO-CCR7+CD28+CD95-CD57+ |
| CD4 stem cell memory (T_SCM_) CD57 | CD45+CD3+CD4+CD45RO-CCR7+CD28+CD95+CD57+ |
| CD4 central memory (TCM) CD57 | CD45+CD3+CD4+CD45RO+CCR7+CD28+CD57+ |
| CD4 transitional memory (TTM) CD57 | CD45+CD3+CD4+CD45RO+CCR7-CD28+CD57+ |
| CD4 effector memory (TEM) CD57 | CD45+CD3+CD4+CD45RO+CCR7-CD28-CD57+ |
| CD4 terminal effector (TTE) CD57 | CD45+CD3+CD4+CD45RO-CCR7-CD28-CD57+ |
| CD8 naïve T cells (TN) CD57 | CD45+CD3+CD8+CD45RO-CCR7+CD28+CD95-CD57+ |
| CD8 stem cell memory (T_SCM_) CD57 | CD45+CD3+CD8+CD45RO-CCR7+CD28+CD95+CD57+ |
| CD8 central memory (TCM) CD57 | CD45+CD3+CD8+CD45RO+CCR7+CD28+CD57+ |
| CD8 transitional memory (TTM) CD57 | CD45+CD3+CD8+CD45RO+CCR7-CD28+CD57+ |
| CD8 effector memory (TEM) CD57 | CD45+CD3+CD8+CD45RO+CCR7-CD28-CD57+ |
| CD8 terminal effector (TTE) CD57 | CD45+CD3+CD8+CD45RO-CCR7-CD28-CD57+ |
| CD4 recent thymic emigrants (RTE) CD57 | CD45+CD3+CD4+CD45RO-CCR7+CD31+CD57+ |
|  |  |
| **KLRG-1 overlay** |  |
| T cells KLRG-1 | CD45+CD3+KLRG-1+ |
| B cells KLRG-1 | CD45+CD19+KLRG-1+ |
| NK cells KLRG-1 | CD45+CD56+KLRG-1+ |
| CD4 T cells KLRG-1 | CD45+CD3+CD4+KLRG-1+ |
| CD8 T cells KLRG-1 | CD45+CD3+CD8+KLRG-1+ |
| Regulatory T cells (TREG) KLRG-1 | CD45+CD3+CD4+FOXP3+CD25+KLRG-1+ |
| CD4 naïve T cells (TN) KLRG-1 | CD45+CD3+CD4+CD45RO-CCR7+CD28+CD95-KLRG-1+ |
| CD4 stem cell memory (T_SCM_) KLRG-1 | CD45+CD3+CD4+CD45RO-CCR7+CD28+CD95+KLRG-1+ |
| CD4 central memory (TCM) KLRG-1 | CD45+CD3+CD4+CD45RO+CCR7+CD28+KLRG-1+ |
| CD4 transitional memory (TTM) KLRG-1 | CD45+CD3+CD4+CD45RO+CCR7-CD28+KLRG-1+ |
| CD4 effector memory (TEM) KLRG-1 | CD45+CD3+CD4+CD45RO+CCR7-CD28-KLRG-1+ |
| CD4 terminal effector (TTE) KLRG-1 | CD45+CD3+CD4+CD45RO-CCR7-CD28-KLRG-1+ |
| CD8 naïve T cells (TN) KLRG-1 | CD45+CD3+CD8+CD45RO-CCR7+CD28+CD95-KLRG-1+ |
| CD8 stem cell memory (T_SCM_) KLRG-1 | CD45+CD3+CD8+CD45RO-CCR7+CD28+CD95+KLRG-1+ |
| CD8 central memory (TCM) KLRG-1 | CD45+CD3+CD8+CD45RO+CCR7+CD28+KLRG-1+ |
| CD8 transitional memory (TTM) KLRG-1 | CD45+CD3+CD8+CD45RO+CCR7-CD28+KLRG-1+ |
| CD8 effector memory (TEM) KLRG-1 | CD45+CD3+CD8+CD45RO+CCR7-CD28-KLRG-1+ |
| CD8 terminal effector (TTE) KLRG-1 | CD45+CD3+CD8+CD45RO-CCR7-CD28-KLRG-1+ |
| CD4 recent thymic emigrants (RTE) KLRG-1 | CD45+CD3+CD4+CD45RO-CCR7+CD31+KLRG-1+ |
|  |  |
| **GZMK overlay** |  |
| T cells GZMK | CD45+CD3+GZMK+ |
| B cells GZMK | CD45+CD19+GZMK+ |
| NK cells GZMK | CD45+CD56+GZMK+ |
| CD4 T cells GZMK | CD45+CD3+CD4+GZMK+ |
| CD8 T cells GZMK | CD45+CD3+CD8+GZMK+ |
| Regulatory T cells (TREG) GZMK | CD45+CD3+CD4+FOXP3+CD25+GZMK+ |
| CD4 naïve T cells (TN) GZMK | CD45+CD3+CD4+CD45RO-CCR7+CD28+CD95-GZMK+ |
| CD4 stem cell memory (T_SCM_) GZMK | CD45+CD3+CD4+CD45RO-CCR7+CD28+CD95+GZMK+ |
| CD4 central memory (TCM) GZMK | CD45+CD3+CD4+CD45RO+CCR7+CD28+GZMK+ |
| CD4 transitional memory (TTM) GZMK | CD45+CD3+CD4+CD45RO+CCR7-CD28+GZMK+ |
| CD4 effector memory (TEM) GZMK | CD45+CD3+CD4+CD45RO+CCR7-CD28-GZMK+ |
| CD4 terminal effector (TTE) GZMK | CD45+CD3+CD4+CD45RO-CCR7-CD28-GZMK+ |
| CD8 naïve T cells (TN) GZMK | CD45+CD3+CD8+CD45RO-CCR7+CD28+CD95-GZMK+ |
| CD8 stem cell memory (T_SCM_) GZMK | CD45+CD3+CD8+CD45RO-CCR7+CD28+CD95+GZMK+ |
| CD8 central memory (TCM) GZMK | CD45+CD3+CD8+CD45RO+CCR7+CD28+GZMK+ |
| CD8 transitional memory (TTM) GZMK | CD45+CD3+CD8+CD45RO+CCR7-CD28+GZMK+ |
| CD8 effector memory (TEM) GZMK | CD45+CD3+CD8+CD45RO+CCR7-CD28-GZMK+ |
| CD8 terminal effector (TTE) GZMK | CD45+CD3+CD8+CD45RO-CCR7-CD28-GZMK+ |
| CD4 recent thymic emigrants (RTE) GZMK | CD45+CD3+CD4+CD45RO-CCR7+CD31+GZMK+ |

**Supplemental Figure 1:** Categorization algorithm for T cell subsets.


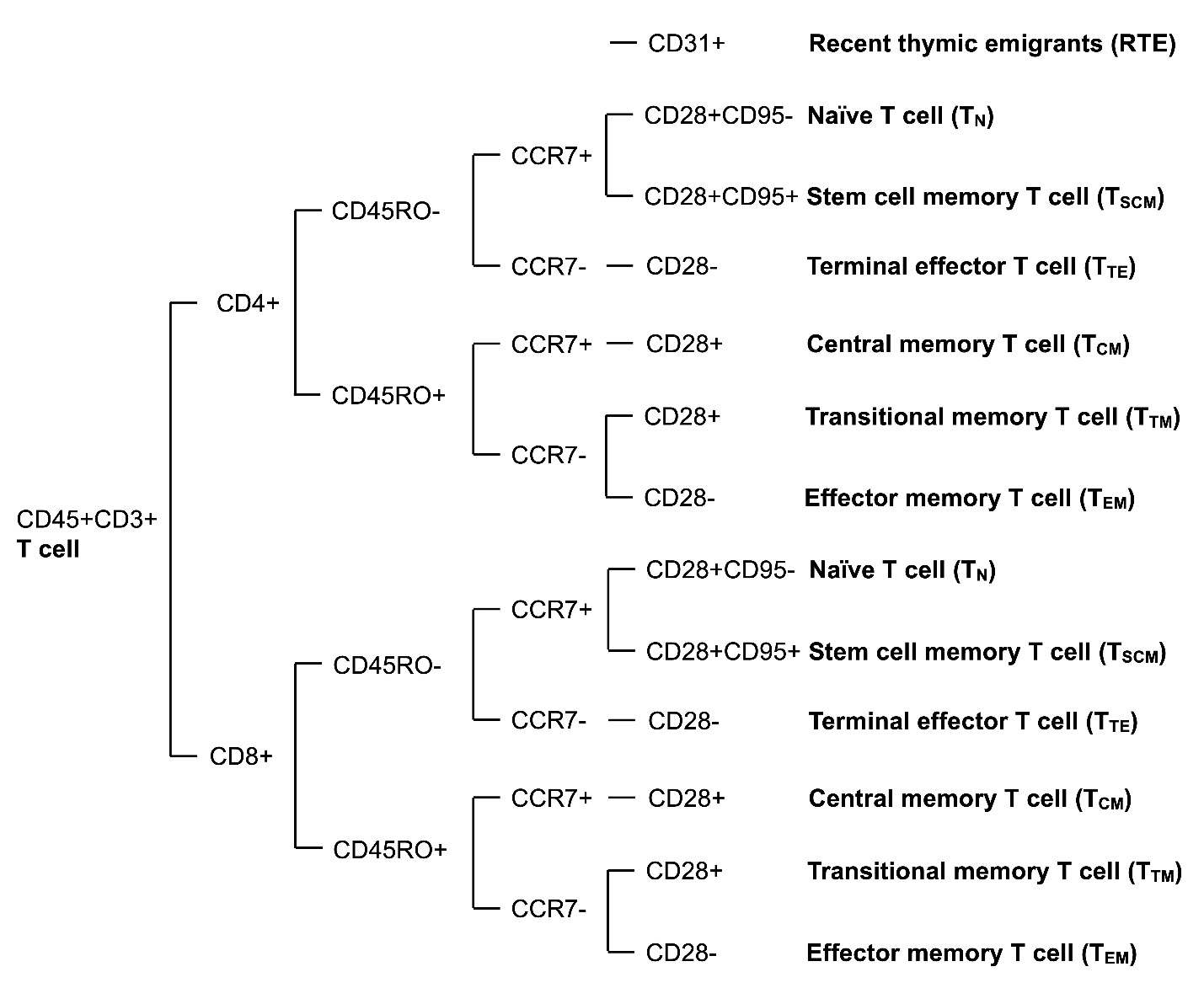


Supplemental **Figure 2.** Scatterplots showing the correlation of lymphocyte counts (x10^6^ cells/ml) with absolute counts (x10^6^ cells/ml) of each studied immune cell phenotypes.


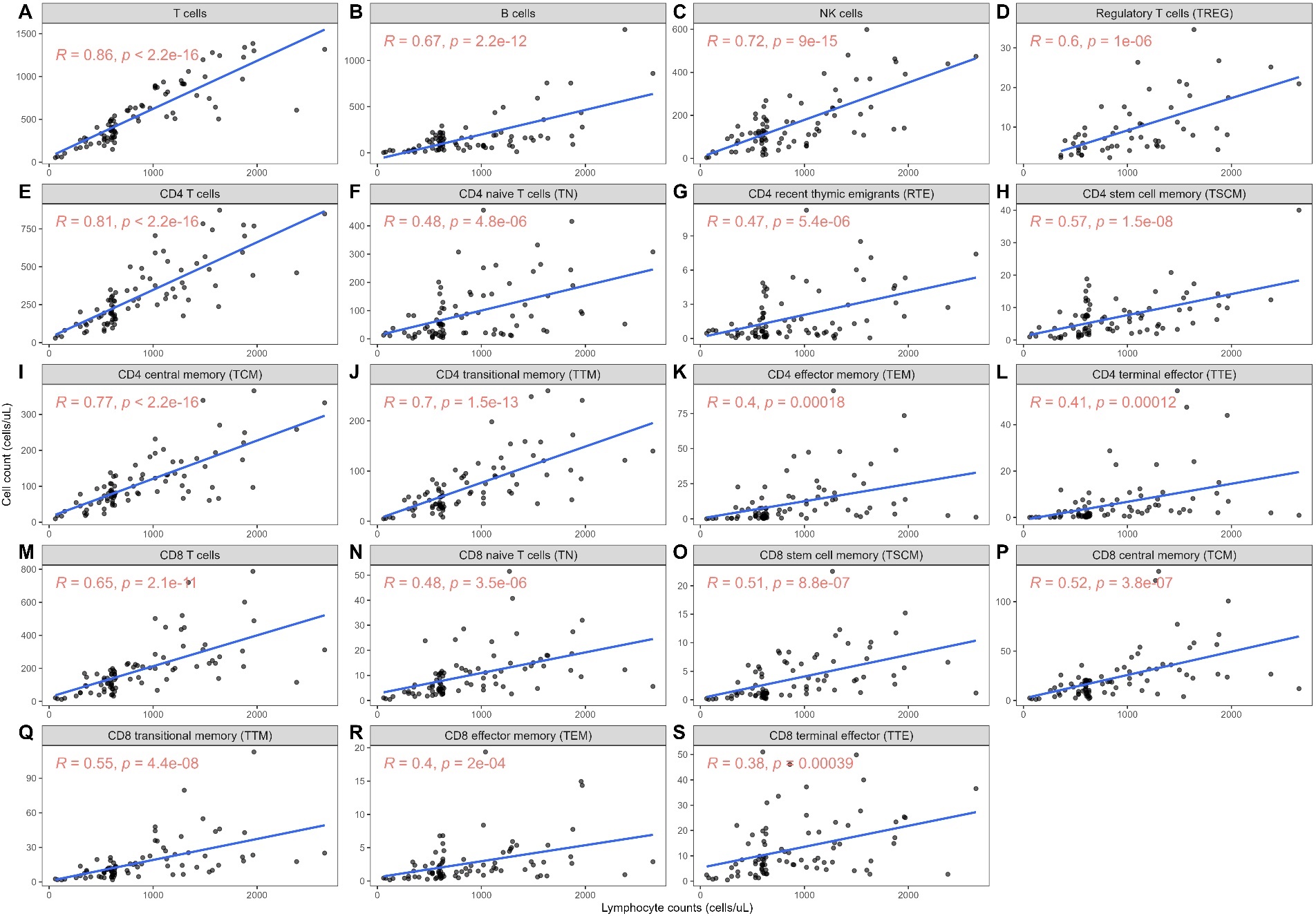


**Supplemental Figure 3*.*** Scatterplots showing the correlation of lymphocyte counts (x10^6^ cells/ml) with proportion of selected CD4+ and CD8+ T cell subsets.


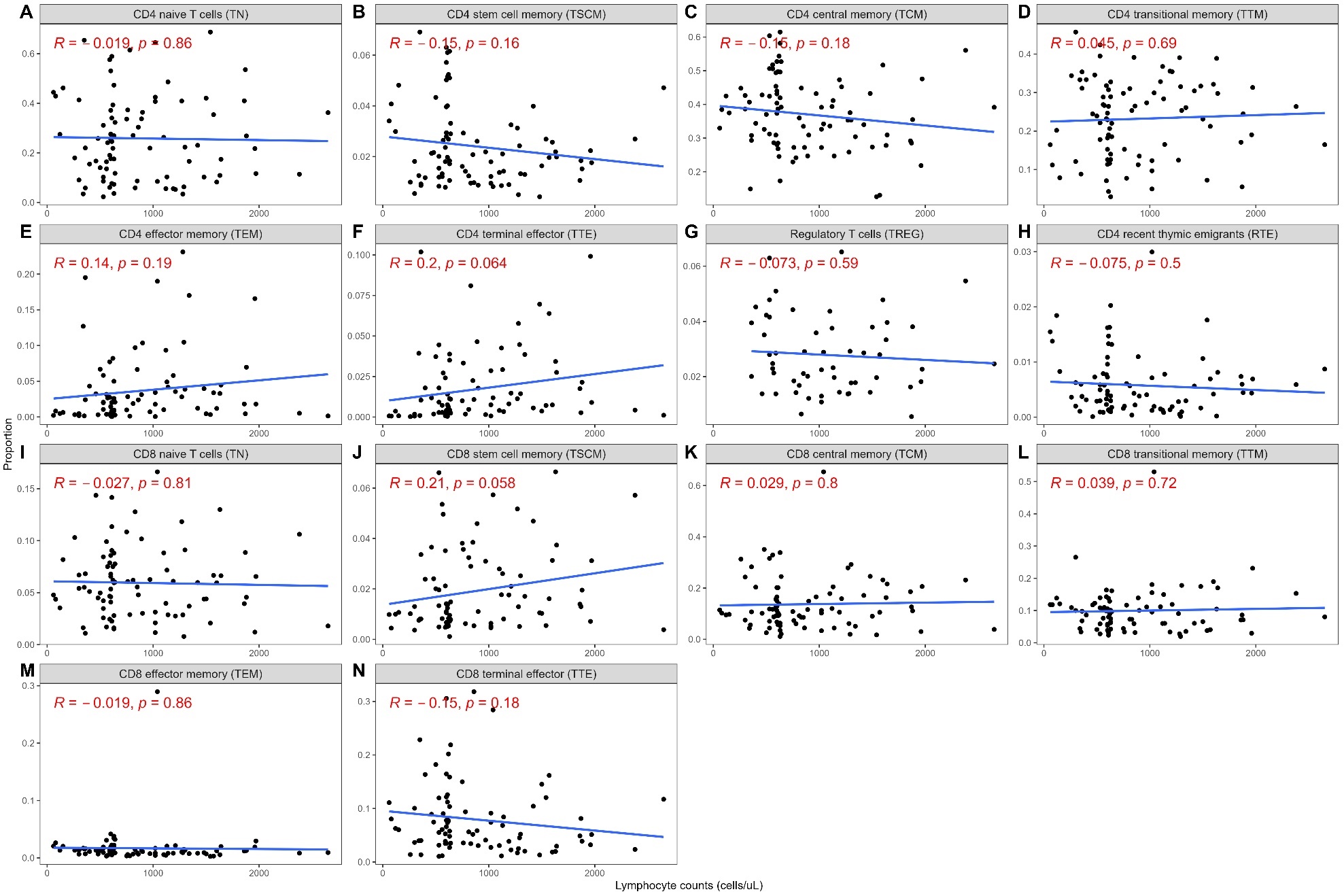


**Supplemental Figure 4*.*** Scatterplots showing the correlation of lymphocyte counts (x10^6^ cells/ml) with proportions of T, B and NK cells expressing functional markers CD57, KLRG-1, PD-1, GZMK, Ki-67.


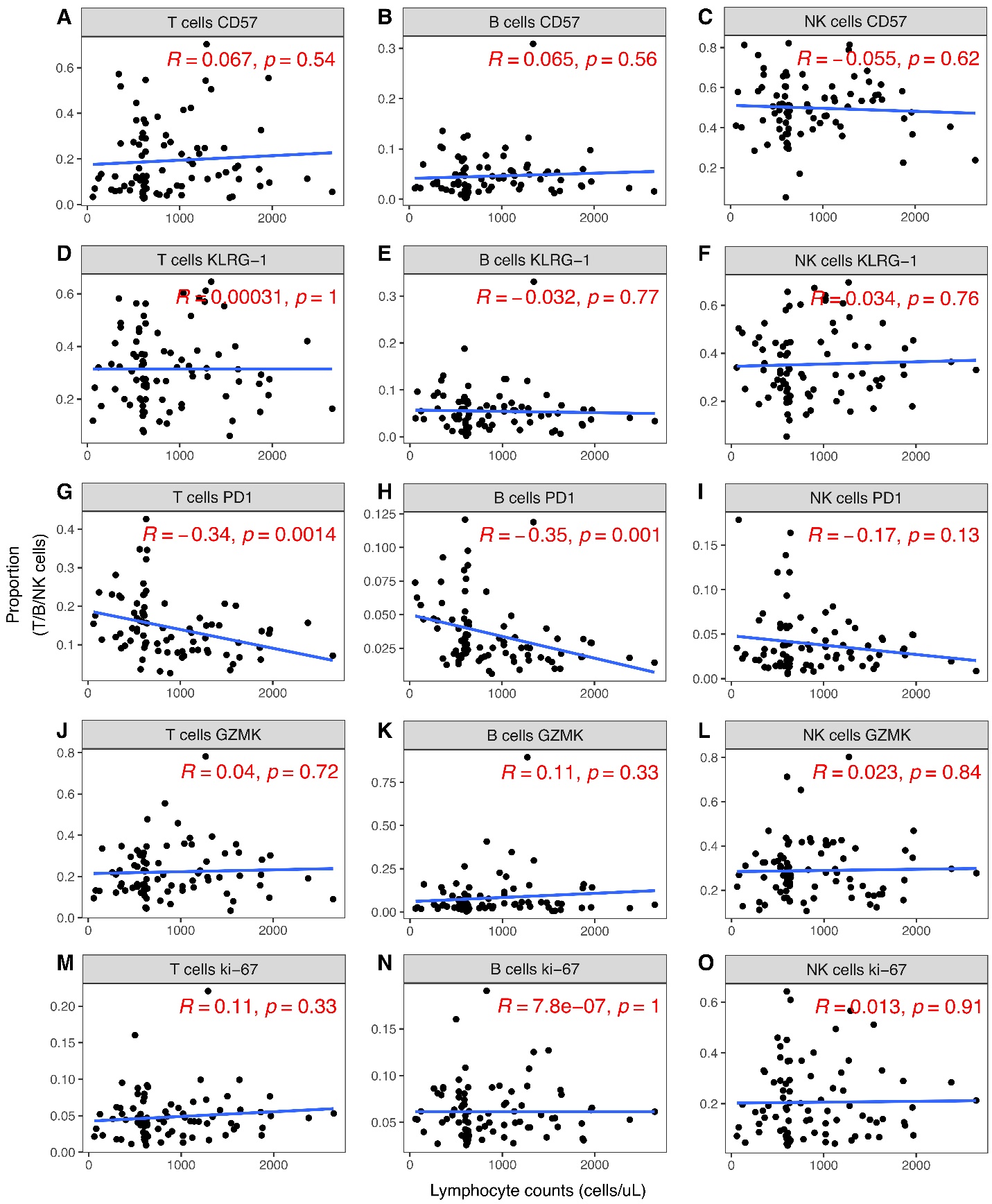


**Supplemental Figure 5.** Scatterplots showing significant correlations of lymphocyte counts (x10^6^ cells/ml) with proportions of CD4+ and CD8+ T cell subsets expressing functional markers.

**
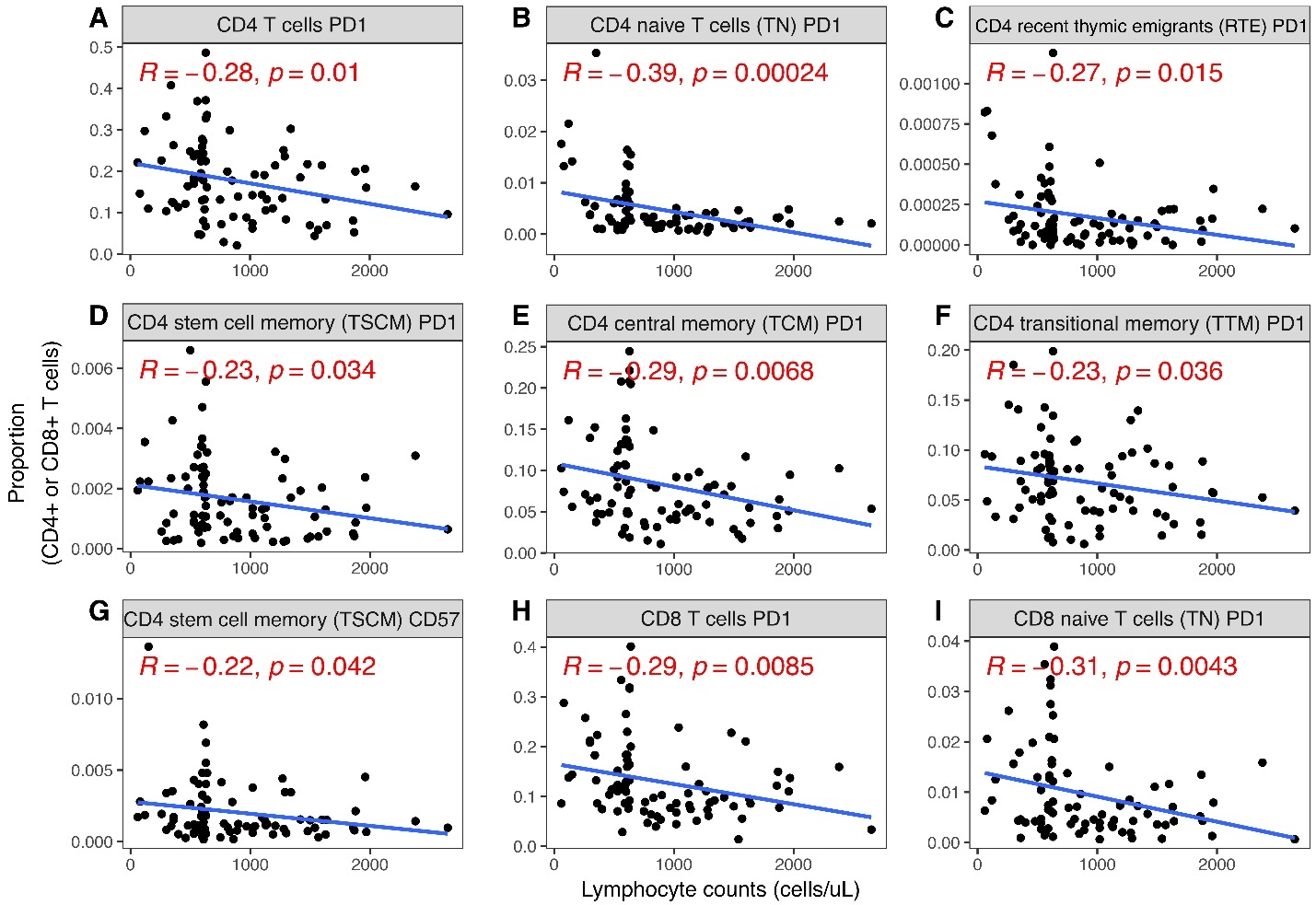
**

**Supplemental Methods**

CMV prophylaxis and induction therapy selection:

The current clinical guideline for cytomegalovirus (CMV) prophylaxis for seronegative recipients who received kidneys from seropositive donors (i.e. CMV R-/D+) at Mayo Clinic is administration of oral valganciclovir 900mg daily for 6months or ganciclovir 5mg/kg IV daily for 6months for recipients who are unable to tolerate enteral medications. After the 6-month period, recipients who are at high risk for developing severe CMV disease will be monitored via weekly CMV PCR testing. Those who are not considered at high risk will undergo routine follow-up clinical visit and CMV PCR testing performed for cause. CMV viremia is defined as detectable levels of CMV on PCR testing while CMV infection is defined as clinical presentation of signs and symptoms associated with CMV as determined by physicians.

For induction therapy selection, most transplant recipients will receive either standard dosage of alemtuzumab or thymoglobulin following transplantation. However, in our clinical practice, recipients who are aged 65 or older or considered to be high risk for development of infection or post-transplant lymphoproliferative disorder are given basiliximab for induction therapy. Following induction, recipients are discharged with standard maintenance immunosuppressive regimen consisting of calcineurin inhibitor, mycophenolate mofetil, and steroid.

*Frozen peripheral blood mononuclear cells (PBMC)*

In addition to routine standard of care laboratory evaluation, peripheral blood was obtained from recipients at time of transplant and at all subsequent routine visits. These peripheral blood samples were processed using standard Ficoll gradient separation at room temperature and the resulting mononuclear cell layer were collected, frozen, and stored in a biobank repository.

Cytometry by Time of Flight (CyTOF)

A random cohort of recipients with at least three years of post-KTx follow up and frozen PBMC samples were selected to undergo immunophenotyping via CyTOF.

*Reagents*

The Maxpar® Direct™ Immune Profiling Assay™ (PN 201325) was used to stain and prepare PBMC for CyTOF. This kit includes Cell Staining Buffer (CSB), Fix and Perm Buffer, PBS, antibodies lyophilized in 5 mL tube (Table 1) and Cell-ID Intercalator-Ir. Cell-ID™ Cisplatin-195Pt (PN 201195), Perm-S solution (201066) and EQ Four Element Calibration Beads (PN 201078) were purchased from Standard BioTools. Human TruStain FcX™ (Fc receptor blocking solution) was purchased from Biolegend (422302). The FoxP3/Transciption Factor Staining Buffer set was purchased from ThermoFisher Scientific. Paraformaldehyde (PFA; 15710) was purchased from EM Sciences and 10X PBS pH 7.2 (MB-008) was purchased from Rockland. The pre-conjugated antibody to PD-1 was purchased from Standard BioTools. The antibodies CD62L, CD95, CD31, Tim-3 and KLRG-1 were purchased from Biolegend. Granzyme-K antibody was purchased from Santa Cruz. Unconjugated antibodies were purchased were custom conjugated in-house through the Mayo Clinic Hybridoma Core using Maxpar X8 or MCP9 antibody labeling kits (Standard BioTools) (see Supplementary Tables 1 and 2 for complete markers and associated phenotypes).

*Samples and Processing*

Peripheral blood mononuclear cells processed and recovered from cryopreservation on the day of preparation for CyTOF. One to 3 million cells were suspended in 1 mL of CSB after recovery from cryopreservation. Each sample was incubated for 5 minutes with a 0.5 um Cisplatin-195Pt solution in PBS. Samples were then centrifuged and washed twice with CSB. After the addition of Fc receptor block, cells were added to a 5 mL tube containing lyophilized antibodies. Upon reconstitution of the pellet additional antibodies in solution were added to the cells. Samples were then incubated at room temperature for 30 minutes. After washing twice with CSB, samples were fixed with 2% PFA in PBS. After fixation and wash, cells were permeabilized using FoxP3/Transcription staining buffer (Thermo-Fisher) and then resuspended in permeabilization solution containing antibodies to intracellular and nuclear markers before incubation at room temperature for 45 minutes. Cells were washed twice with CSB and then resuspended in 30 nM intercalation solution and incubated at 4 ºC on a rocker overnight. To facilitate batch variation a replicate reference PBMC sample derived from apheresis cones prepared with samples on each staining day. Samples were prepared in batches of 7 to 8 samples. After overnight incubation with intercalation solution samples were washed and resuspended in cell acquisition solution containing a 1:10 dilution of EQ four element calibration beads. Prior to data acquisition samples were filtered through a 35 um blue cap tube (Falcon; 35209).

*Mass Cytometry and Data Acquisition*

Samples were loaded onto a Helios CyTOF® system (Standard BioTools) using an attached autosampler and were acquired at a rate of 200-400 events per second. Data were collected as .FCS files using the CyTOF software and after acquisition intra file signal drift was normalized to the acquired calibration bead signal using the normalization algorithm embedded in the CyTOF software (version 7.05189.0; Standard BioTools).

*CyTOF data analysis*

FCS files were demultiplexed and normalized to account for drift in marker expression over time. Marker distributions between batches were normalized using the R package CytoNorm (https://github.com/saeyslab/CytoNorm), training the model on technical replicates of a reference sample run in each batch. For each marker and for each sample, the threshold for positive/negative marker expression was calculated by summarizing expression as histogram bins and using algorithm to identify peaks and valleys. The first valley from negative expression represented the threshold between positive (denoted +) and negatively (denoted -) expressing cells. Removal of multiplets, dead cells, and debris was performed by selecting for Beads-, Pt195-, and the largest population of the Bayesian metrics (Offset, Width, Event Length) and DNA1/DNA2.

To visualize the gated cell populations, samples were down-sampled to a uniform 20,000 cells per sample. Cell phenotypes were determined based on the marker panel detailed in Supplemental Table 3, with T cell subset identification following the algorithm outlined in Supplemental Figure 1. Absolute cell counts were derived by applying the proportional representation of each phenotype within the total lymphocyte count (defined as the sum of T, B, and NK cells) to the recipient's lymphocyte count at 3 years post-transplantation. Phenotypic proportions are expressed as percentages relative to their respective parental cell populations (T, B, NK, CD4+ T, or CD8+ T cells).
